# Importance of mechanical cues in regulating musculoskeletal circadian clock rhythmicity: Implications for articular cartilage

**DOI:** 10.14814/phy2.15780

**Published:** 2023-08-03

**Authors:** Lekau Dintwa, Clare E. Hughes, Emma J. Blain

**Affiliations:** ^1^ Biomedicine Division, School of Biosciences Cardiff University Cardiff UK; ^2^ Biomechanics and Bioengineering Centre Versus Arthritis, School of Biosciences Cardiff University Cardiff UK

**Keywords:** articular cartilage, circadian rhythm, extracellular matrix topography, mechanical stimuli, molecular clock, musculoskeletal tissues, osteoarthritis (OA)

## Abstract

The circadian clock, a collection of endogenous cellular oscillators with an approximate 24‐h cycle, involves autoregulatory transcriptional/translational feedback loops to enable synchronization within the body. Circadian rhythmicity is controlled by a master clock situated in the hypothalamus; however, peripheral tissues are also under the control of autonomous clocks which are coordinated by the master clock to regulate physiological processes. Although light is the primary signal required to entrain the body to the external day, non‐photic zeitgeber including exercise also entrains circadian rhythmicity. Cellular mechano‐sensing is imperative for functionality of physiological systems including musculoskeletal tissues. Over the last decade, mechano‐regulation of circadian rhythmicity in skeletal muscle, intervertebral disc, and bone has been demonstrated to impact tissue homeostasis. In contrast, few publications exist characterizing the influence of mechanical loading on the circadian rhythm in articular cartilage, a musculoskeletal tissue in which loading is imperative for function; importantly, a dysregulated cartilage clock contributes to development of osteoarthritis. Hence, this review summarizes the literature on mechano‐regulation of circadian clocks in musculoskeletal tissues and infers on their collective importance in understanding the circadian clock and its synchronicity for articular cartilage mechanobiology.

## 1 INTRODUCTION

Most physiological processes in light‐sensitive organisms are controlled by the circadian clock, a collection of endogenous cellular oscillators with an approximate 24‐h rhythmic cycle, ensuring synchronization to daily variations of light and temperature (Reppert & Weaver, 2002; Roenneberg & Merrow, 2005). Mammalian circadian clock oscillators are ubiquitous and autonomous functioning at a cellular, tissue, and systems level (Mohawk et al., 2012). Robustness of circadian rhythms deteriorate during aging and disease resulting in disturbance in the temporal control of physiology (Nakamura et al., 2011, Orozco‐Solis & Sassone‐Corsi, 2014). The mammalian clock consists of the master clock situated in the suprachiasmatic nucleus (SCN) of the anterior hypothalamus and subordinate clocks found in peripheral tissues (Jacob et al., 2020). Light is the primary signal used by the SCN to entrain the body to the external day. Circadian rhythms can also be entrained by non‐photic zeitgeber including sleep–wake cycle, temperature, and of relevance to this review exercise (Mohawk et al., 2012).

Functionality of physiological systems including cardiovascular, nervous, and musculoskeletal systems rely extensively on their ability to respond to mechanical loading including fluid flow, compression and tensile strain, and adapt their cellular behaviors to elicit appropriate responses. Mechanical loading is involved in regulating circadian rhythmicity in tissues including skeletal muscle (Bae et al., 2006; Saracino et al., 2019; Sasaki et al., 2016; Vanmunster et al., 2022; Wang et al., 2021; Wolff & Esser, 2012; Yamanaka et al., 2008), intervertebral disc (Ding et al., 2021), bone (Bouchard et al., 2022), and cartilage (Gossan et al., 2013; Heywood et al., 2022; Kanbe et al., 2006) which can impact tissue homeostasis. Specifically, extrinsic mechano‐regulation of circadian rhythms can promote extracellular matrix (ECM) synthesis; however, circadian dysregulation results in tissue catabolism and onset of pathology (Dudek et al., 2016; Guo et al., 2015; Kc et al., 2015; Snelling et al., 2016). Equally, the intrinsic ECM environment defining the material properties of a tissue (stiffness and elasticity) can influence the circadian clock (Yang et al., 2017) (Williams et al., 2018).

### 1.1 Molecular mechanism of the mammalian circadian clock

**FIGURE 1:**
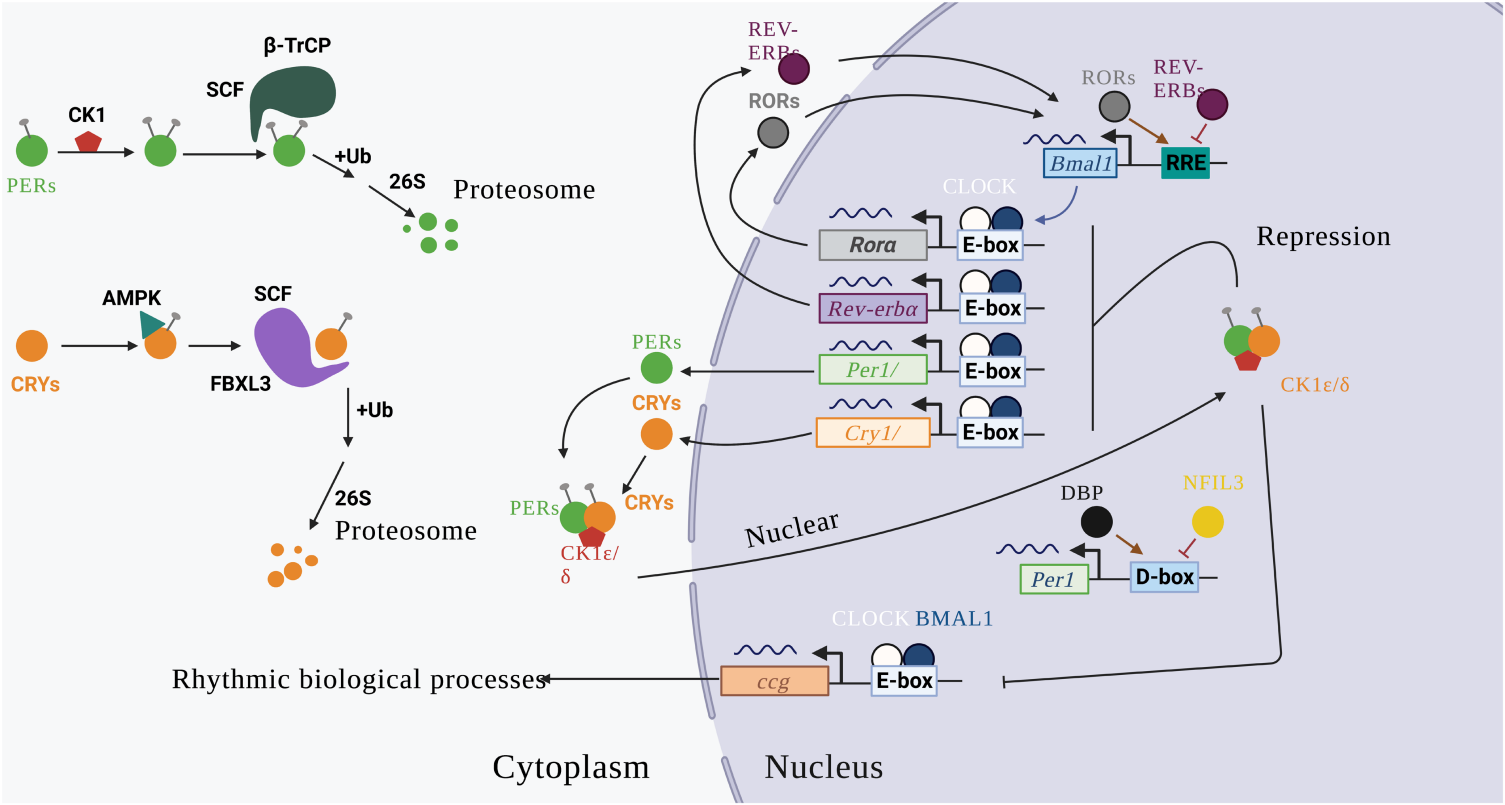
Transcriptional/translational feedback loops of mammalian clock genes and proteins driving rhythmic circadian oscillations intracellularly. Upon the activation of *Bmal1* (brain and muscle ARNT‐Like 1/ aryl hydrocarbon receptor nuclear translocator‐like protein 1 (*ARNTL*)) transcription by the binding of RORs (retinoic acid receptor‐related orphan receptor) to Rev‐responsive element (RRE) in its promoters, *Bmal1* heterodimerize with Clock (circadian locomotor output cycles protein kaput) forming *Bmal1*/Clock complexes which activates the transcription of their target genes such as *Pers* (period circadian regulator −1 and − 2), *Crys* (cryptochrome circadian regulator −1 and −2), *Rors* (Rorα, Rorβ & Rorγ), and *Rev‐erbs* (*−α & β* ‐ reverse strand of *ERB*/*THR‐A and ‐B* [thyroid hormone receptor‐α and ‐β]) by binding to E‐box (enhancer box) regulatory elements in their promotors. Proteins of clock‐controlled genes of the PAR‐bZip family such as *Dbp* (albumin D box‐binding protein) binds to the D‐box (DNA cis‐element box) regulatory elements of core clock genes (e.g., *Per1*) to activate their expression and can be repressed by the bZip protein, Nfil3/E4BP4 (E4 Promoter‐Binding Protein 4) in an auxiliary stabilizing loops. Other additional loops involve genes such as *Bhlhe‐40* and ‐*41* (basic helix–loop–helix family member proteins E‐40 and ‐41), *Timeless* (timeless circadian regulator), *Hlf* (hepatic leukemia factor), and *Tef* (thyrotroph embryonic factor). *Pers* and *Crys* mRNA transcripts are translated in the cytoplasm during the day before being translocated to the nucleus in the evening to repress *Bmal1*/Clock complex induced transcription activation and the remaining proteins are phosphorylated by casein kinase Iε/δ (CK Iε/δ) and AMP kinase (AMPK) for Pers and Crys, respectively. This fosters polyubiquitination by Skp1‐Cullin‐F‐box protein (SCF) E3 ubiquitin ligase complexes involving FBXL3 and β‐TrCP for Crys and Pers before they are degraded by the 26S proteasome complex. The *Bmal1*/Clock complex also binds to the E‐box elements in the promoters of clock controlled/output genes (CCGs) responsible for the regulation of rhythmic biological processes (Created in Biorender with information from Mohawk et al. (2012)).

The core clock mechanism involves interlinking autoregulatory transcriptional/translational feedback loops of clock genes and proteins which drive rhythmic circadian oscillators (Figure 1) (reviewed in Mohawk et al., 2012, Reppert & Weaver, 2002, Roenneberg & Merrow, 2005). Briefly, the primary negative feedback loop consists of the BMAL1/CLOCK heterodimer complex which activates transcription of *Cry1* and *2*, and *Per1* and *2* (Gallego & Virshup, 2007; Jacob et al., 2020; Mohawk et al., 2012). PER/CRY heterodimerize in the evening prior to nuclear translocation where they repress their own transcription (McClung, 2007); PER/CRY are subsequently targeted for polyubiquitination enabling the BMAL1/CLOCK complex to start another cycle of transcriptional activation (Partch et al., 2014; Takahashi et al., 2008). Additional feedback loops including stabilizing loops (involving RORα, β and γ, *NR1D1*, and *NR1D2* [also referred to as *REV‐ERBs])* and auxiliary loops (involving *BHLHE40* and *BHLHE41*, *TIMELESS*, *E4BP4*, *DBP*, *HLF*, and *TEF*) exist which also engage the core CLOCK‐BMAL1/PER‐CRY feedback loop to fine‐tune precision of the clock (Gachon, 2007; Lowrey & Takahashi, 2004; Schroeder & Colwell, 2013; Takahashi et al., 2008; Figure 1). Clock transcription factors also regulate expression of clock‐controlled genes (CCGs)/output genes which are fundamental in driving daily rhythmicity (Reppert & Weaver, 2002; Schibler, 2007; Yang & Meng, 2016).

### 1.2 Mechanical stimuli regulate the circadian rhythm in musculoskeletal tissues

Physiological loads regulate cell and tissue behaviors, with non‐physiological, abnormal loading detrimentally impacting on responses. Mechanical stimulation regulates the circadian clock in musculoskeletal tissues including muscle (Bae et al., 2006; Saracino et al., 2019; Sasaki et al., 2016; Vanmunster et al., 2022; Wang et al., 2021; Wolff & Esser, 2012; Yamanaka et al., 2008), periodontal ligament (Qin et al., 2019), intervertebral disc (Ding et al., 2021), and bone (Bouchard et al., 2022). The first evidence implicating mechano‐regulation of the circadian rhythm was reported in *Drosophila melanogaster* exposed to cycles of 12 h of vibration followed by 12 h of silence (Simoni et al., 2014). These cycles of vibration and silence were sufficient for flies to synchronize their daily locomotor activity; furthermore, *Drosophila* containing a *Per* loss‐of‐function mutation lacked anticipatory behavior (Simoni et al., 2014). Vibration‐induced alterations in the phase of the molecular oscillation in the *Drosophila* chordotonal organ clocks implicated mechanical stimulation as a zeitgeber. Subsequent studies have also demonstrated a link between mechanical stimulation and circadian rhythms in other tissues.

#### 1.2.1 Influence of exercise regimes and compressive load on circadian rhythmicity

##### Skeletal muscle

A seminal study utilizing *Per1*‐luc transgenic mice demonstrated that exposure to an 8 h phase advance in the light–dark cycle fully re‐entrained the skeletal muscle clock in locomotor activity following exercise which was not observed in control mice not exposed to exercise (Yamanaka et al., 2008). However, a phase delay in the light–dark cycle of 8 h led to a significant phase shift in *Per1* reporter rhythm which was independent of exercise. Wolff and Esser (2012) also demonstrated that 2 h of voluntary or forced exercise per day for 4 weeks resulted in a phase shift in skeletal muscle (Wolff & Esser, 2012); interestingly, exercise did not affect the central SCN clock suggesting loading regulates synchronicity of peripheral tissue clocks only. In skeletal muscle, substantially increased *Bmal1* transcript levels was observed in response to acute aerobic activity (treadmill exercise) whereas acute high force muscle contractions induced *Per1* and *Per2* transcription, indicating that different loading regimens can elicit divergent clock responses. Mechano‐regulation of the skeletal muscle clock has more recently been postulated to be partially dependent upon glucocorticoid signaling as REDD1^−/−^ mice (Regulated in Development and DNA Damage 1) exposed to aerobic exercise did not induce *Per1* expression (Saracino et al., 2019); it has been hypothesized that induction of *Per1* expression may contribute to an increase in skeletal muscle work capacity, particularly as *Per2* deletion has been previously found to reduce exercise capacity (Bae et al., 2006). Exercise responsive adrenal hormones, aldosterone and epinephrine have also been implicated in regulating the core clock in a REDD1‐dependent manner (Saracino et al., 2019); this might implicate cellular stress in indirectly regulating peripheral clocks through the production of stress hormones and elevated glucocorticoid signaling following sustained exercise.

##### Bone

Surprisingly, only one study has been reported to date on the involvement of circadian oscillations in driving compression induced responses in bone. In this in vivo murine tibial loading study, cyclic compressive loading (11 N, 4 Hz, 216 cycles/day) was applied at ZT2 (light phase) or ZT14 (dark phase) for a single episode or for 2 weeks (Bouchard et al., 2022). Although compressive loading did not significantly regulate *Bmal1*, *Clock*, *Per1*, and *Per2* transcription following a single loading episode, several downstream clock‐controlled genes were differentially regulated, depending on the zeitgeber time; in mouse cortical bone loaded at ZT14, the mechanosensitive gene *Sost* was significantly decreased 1 and 24 h post‐load whereas in mice loaded at ZT2, its expression was significantly increased 8 h post‐loading. Transcription of the mechanosensitive *Dkk1* was also decreased at 1 h post‐loading at ZT14, whereas increases were observed after 8 h in mice loaded at ZT2. Osteocyte markers were also differentially regulated according to the ZT time of loading including *Runx2* and *Bglap* (osteocalcin) (ZT2, 24 h post‐load), *Ctsk* (cathepsin K; ZT14, 8 h post‐load), and *Tnfrsf11b* (osteoprotegerin) (ZT2 and 14, 8 h post‐load) (Bouchard et al., 2022). Interestingly, greater cortical bone formation was observed at ZT14 (compared to ZT2) in the midshaft region; bone remodeling was also found to be impacted more by night loading with increased periosteal resorption in mice loaded at ZT14 (Bouchard et al., 2022). Analyses also indicated that the circadian effects on load‐induced bone remodeling were site‐specific, that is effects were not observed in the metaphyseal cortical nor trabecular bone, although it was speculated that these site‐specific differences might be attributed to differences in local tissue strains, fluid flow, or sclerostin abundance (Bouchard et al., 2022).

#### 1.2.2 Influence of tensile strain on circadian rhythmicity

##### Muscle

In a C2C12 myoblast cell line subjected to 15% cyclic strain (0.5 Hz, 12 h), *Clock* and *Bmal1* transcription were markedly increased with decreased *Per* and *Cry* transcription (Wang et al., 2021). After 24 h, *Clock* and *Bmal1* transcript levels were significantly decreased, concomitant with significantly elevated *Per* and *Cry* mRNA, suggestive that strain per s and loading duration induce a distinct skeletal muscle clock response (Wang et al., 2021).

##### Periodontal ligament

Equi‐biaxial strain (12%, 6 s every 90 s, 4 h) synchronized the circadian rhythmicity of osteogenic genes in periodontal ligament fibroblasts, with transcription of type I collagen *(COL1A1)*, osteopontin *(OPN)*, and integrin‐binding sialoprotein *(IBSP)* concomitant with the light/dark rhythmic cycle of *Per1* expression (Qin et al., 2019). Furthermore, in vivo application of orthodontic force to rat teeth (30 g/cm^2^ force, 2 weeks) synchronized the clock inducing significant circadian oscillations in *Col1A1*, *OPN*, and *IBSP* gene expression suggestive that mechanical cues can elicit a clock‐dependent osteogenic response (Qin et al., 2019).

##### Intervertebral disc

In rat intervertebral disc (IVD), nucleus pulposus (NP) cells subjected to 10% strain (0.5 Hz, 24 h) increased *Bmal1* and *Per1* up to 24 h after strain cessation (Ding et al., 2021). In contrast, higher strains (18%, 0.2 Hz, 24 h) reduced the oscillation amplitude of *Bmal1* and *Clock* mRNA levels after synchronization; BMAL1 and CLOCK protein levels were also significantly reduced suggesting that application of excessive strain disrupts the NP clock. Interestingly, RNA sequencing and enrichment analysis of degenerated NP tissue identified genes associated with F‐actin reorganization (Ding et al., 2021); inhibition of actin polymerization reversed the oscillation amplitude of *Bmal 1* expression in NP cells exposed to 18% strain, a mechanism hypothesized to be mediated via the Rho/ROCK pathway. Parallel in vivo studies, performed using a rat model of prolonged upright posture to imitate human IVD loading, demonstrated that shifting the light–dark cycle to mimic the environmental cues of night shift workers led to significantly reduced proteoglycan content (Ding et al., 2021). An important implication of this study's findings is that disturbance of the IVD's intrinsic circadian rhythm due to night shift occupations, combined with altered/excessive loading, could contribute to IVD degeneration (Ding et al., 2021).

#### 1.2.3 Influence of unloading on circadian rhythmicity

The effect of unloading (as a model of non‐physiological loading) on gastrocnemius muscle, investigated in mice via hindlimb suspension for 14 days, resulted in increased *Cry2* and *Per2* transcription with no effect on *Bmal1*, *Clock*, *RevErba*, *RevErbb*, and *Per1* expression (Vanmunster et al., 2022); this suggests that mRNA expression of transcriptional activators are unaffected by unloading, whereas transcriptional repressors are mechanically regulated in fast‐twitch muscles. In contrast, *Bmal1*, *Clock*, *RevErba*, and *RevErbb* expression levels were suppressed in soleus muscles following hindlimb suspension, suggesting that unloading elicits divergent clock responses in fast‐twitch and slow‐twitch muscles fibers (Vanmunster et al., 2022). Strong positive correlations between identified mechano‐sensors (*Ilk1* ‐ integrin‐linked kinase, *Fermt2* ‐ kindlin‐2) and core clock genes (*Clock* and *Bmal1*) in both unloaded and control gastrocnemius and soleus muscles suggested a regulatory connection between mechano‐sensing and molecular clock activity in skeletal muscle tissues (Vanmunster et al., 2022). *Ilk1* and *Fermt2* knockdown in C2C12 myotubes significantly reduced expression levels of mechano‐sensor, molecular clock, and metabolism related‐ genes, further demonstrating the regulation of skeletal muscle clock machinery by core mechano‐sensors (Vanmunster et al., 2022).

### 1.3 Influence of the cellular mechano‐environment on circadian rhythmicity

The tissues' ECM provides a biomechanical environment in which its material properties, for example stiffness resulting from increased ECM deposition and/or crosslinking, can influence cell behaviors (Gilbert et al., 2021; Liao et al., 2023). ECM topography including the nano‐scale alignment of protein fibrils and macro‐scale ECM architecture including shape, organization, and geometry directly influence the cellular mechano‐environment (Bai et al., 2020). Although not conducted in musculoskeletal tissues to date, studies in ex vivo 3D versus 2D mammary epithelial cell (MEC) models revealed that the PER2 amplitude in 3D cultured cells was significantly stronger than its 2D equivalent when exposed to an identical mechanical stiffness (Yang et al., 2017); a stronger rhythmic expression of *Per2* and *Nr1d1* mRNA levels as well as clock target genes were also observed in 3D compared to 2D culture. Interestingly, at the single cell level, individual MECs seeded at low density in 3D culture (stiffness of 30 Pa) generated a higher clock amplitude compared to equivalent 2D cultured individual cells (stiffness >100 MPa) suggesting that the extracellular micro‐environment regulates the strength of epithelial circadian activity (Yang et al., 2017); higher amplitude oscillations (and magnitude in most cases) were also observed in keratinocytes embedded in softer 3D matrices (Williams et al., 2018). However, other studies have demonstrated an inverse relationship between matrix stiffness, geometry, and clock behavior with stronger clock activities in mammary and lung epithelial cells and dermal fibroblasts cultured in stiffer 2D matrices compared to softer 3D microenvironments (Williams et al., 2018). Interestingly, clock activities were not modulated in MECs cultured on 2D‐substrata coated with different ECM proteins, providing further evidence that peripheral clock activity is regulated by the stiffness of the ECM microenvironment rather than composition (Yang et al., 2017).

### 1.4 Articular cartilage composition and biomechanical functionality

Articular cartilage, covering the ends of diarthrodial joints, has a unique biochemical composition facilitating dissipation of mechanical forces and ensuring smooth articulation of joints. These biomechanical properties are conferred by its ECM composition and organization, specifically a high density of proteoglycans embedded within a collagenous network that provides mechanical resilience (comprehensively reviewed in Gilbert et al., 2021). However, the spatial organization of these ECM molecules is also pivotal in supporting load dissipation and facilitating mechano‐sensing that is, detecting mechanical perturbations in the extracellular environment and effecting cellular responses, by the sole cell type, the chondrocyte. Chondrocytes sense alterations in the magnitude or type of loading stimulus applied inducing changes in cellular behaviors. In situ, chondrocytes experience a combination of compression, hydrostatic pressure, shear stress, osmotic stress, and tensile strain. Exposure to physiological loading induces a homeostatic balance of ECM biosynthesis coupled with catabolic activities to ensure a slow, but consistent turnover of the cartilage. In contrast, non‐physiological loading (typified by excessive loading, joint malalignment or traumatic injury) disrupts this homeostatic balance favoring elevated catabolism, cartilage degeneration, and osteoarthritis (OA) progression. A complex interplay of loading, inflammation, and growth factors also impacts the biochemical and biomechanical hierarchy of articular cartilage.

#### 1.4.1 Articular cartilage possesses an autonomous clock rhythmicity

A time‐dependent expression of core clock genes (*Bmal1*, *Cry1*, *Nr1d1*, and *Per2*) was observed in chondrocytes with a periodicity of ~24.5 h demonstrating that chondrocytes harbor cell autonomous circadian clocks (Gossan et al., 2013). In healthy murine cartilage, *Cry1* expression maintained a periodicity of ~24.3 ± 3 h (Guo et al., 2015) and expression of *Bmal1*‐regulated genes (*Rev‐Erbα* and *Per2*) were also time‐dependent (Dudek et al., 2016), providing further evidence of autonomous clock rhythmicity. Spontaneous and robust oscillations in *BMAL1* and *NR1D1* transcript and protein levels were also reported in healthy human knee chondrocytes (Akagi et al., 2017) further verifying that chondrocytes possess functional and self‐sustained circadian clocks.

##### Influence of mechanical stimulation

Surprisingly, considering the biomechanical function of articular cartilage in vivo, very few studies to date have considered the impact of loading on regulating the cartilage clock. However, Kanbe et al. (2006) demonstrated that *Clock* is a mechanosensitive gene, as transcript levels were significantly downregulated following exposure of mouse chondrocytes to tensile strain (5%, 1 Hz, 15 min/h, 4 days) in a 3D sponge model (Kanbe et al., 2006). Heywood et al. (2022) recently demonstrated that daily episodes of tensile strain (10%, 0.33 Hz, 12 h) synchronized the chondrocyte clock over 3 days with BMAL‐1 periodicity aligning with diurnal mechanostimulation (Heywood et al., 2022); introduction of a 6 h phase shift in loading resulted in an equivalent shift of the cartilage clock. Furthermore, *Bmal1*, *Per2/3*, *Cry1*, and *Rev‐erb* transcription was reported to be induced in embryonic chick limb bud‐derived chondroprogenitor cell micromass cultures subjected to compression (0.6 kPa, 0.05 Hz, 1 h daily, 6 days) concomitant with rhythmic expression of *Sox6*, *Sox9*, and *Acan* mRNAs (Vago et al., 2022), indicating that mechanical stimulation can induce chondrogenesis via a clock‐dependent mechanism. Interestingly, elevated *Bmal1* mRNA levels concomitant with significant dysregulation of clock output genes (*Nr1d1* and *Dbp* decreased; *Rora* and *E4bp4* increased) were observed in a murine model of OA joint instability (medial meniscus destabilization or DMM model) (Gossan et al., 2013); thus, abnormal mechanical loading may directly contribute to tissue degeneration via a cartilage clock‐dependent mechanism.

#### 1.4.2 Disruption of cartilage clock promotes development of an OA phenotype

An emerging role for the “cartilage clock” in modulating tissue homeostasis has implications for matrix degeneration and OA development, as reduced dexterity, pain sensation, and stiffness in knees of OA patients manifests in a circadian pattern (Bellamy et al., 1990). Furthermore, significantly higher *PER2* mRNA levels concomitant with reduced *BMAL1* expression was evident in human OA cartilage indicating disruption of the chondrocytes' intrinsic clock compared to undamaged cartilage (Snelling et al., 2016). *BMAL1* knockdown induced cell proliferation and *MMP13* transcription in healthy chondrocytes recapitulating the OA chondrocyte phenotype suggesting *BMAL1* potentially contributes to maintaining the chondrocyte phenotype in human cartilage and that reduced *BMAL1* expression may contribute to early OA pathogenesis (Snelling et al., 2016). “Circadian rhythm” was the most dysregulated pathway in human OA cartilage with significantly modulated expression of core clock, stabilizing and auxiliary loop genes compared to healthy cartilage (Akagi et al., 2017). TGF‐β signaling was significantly impacted following chondrocyte *BMAL1* and *NR1D1* suppression (Akagi et al., 2017), providing a mechanistic link between a dysregulated circadian clock and aberrant TGF‐β signaling (pivotal in cartilage homeostasis) in creating a biosynthetic shift toward tissue catabolism.

Environmental disruption of circadian rhythms, induced by weekly 12 h phase shifts in the LD cycle to replicate night shift occupations, significantly reduced murine knee cartilage proteoglycan content concomitant with fibrillation and mast cell infiltration of the synovium over 22 weeks (Kc et al., 2015). Perhaps surprisingly, genetically perturbing the circadian clock machinery using *Clock* and *tau* mutant mice did not induce spontaneous joint pathology, suggesting that environmental disruption of circadian rhythms per se is a key driver of OA pathology (Kc et al., 2015). However, chondrocyte specific conditional *Bmal1* ^*−/−*^ mice demonstrated progressive cartilage degeneration with age (Dudek et al., 2016). Genes associated with TGF‐β(p‐SMAD2/3, p‐SMAD1/5) and NFAT (NFATC2) signaling were identified as significant upstream regulators in the *Bmal1* ^*−/−*^ cartilage culminating in reduced expression of chondrocyte‐specific genes (Dudek et al., 2016). This provides direct evidence implicating circadian disruption as a potential OA risk factor reducing anabolism and activating catabolic/apoptotic pathways in cartilage (Dudek et al., 2016).

In vivo evidence not only implicates clock disruption in cartilage degeneration but also in predisposing the joint to inflammation. Circadian rhythm was dampened in ex vivo cartilage explants exposed to the pro‐inflammatory cytokine interleukin‐1 (IL‐1), concomitant with a reduction in *Cry1*, *Cry2*, *Dbp*, *Nr1d1*, *Per1*, and *Per2* mRNA levels and elevated transcription of genes involved in cartilage catabolism. IL‐1β may disrupt the function of the core clock mechanism by reducing the ability of CLOCK/BMAL1 to transactivate E‐box promoters (Guo et al., 2015). Interestingly, exposure to tumor necrosis factor alpha (TNFα) had minimal effect on circadian rhythmicity suggesting that different cytokines elicit divergent influences on cartilage clocks and highlights the potential of the cartilage clock as a novel target for joint inflammation (Guo et al., 2015).

### 1.5 Future directions and concluding remarks

**FIGURE 2:**
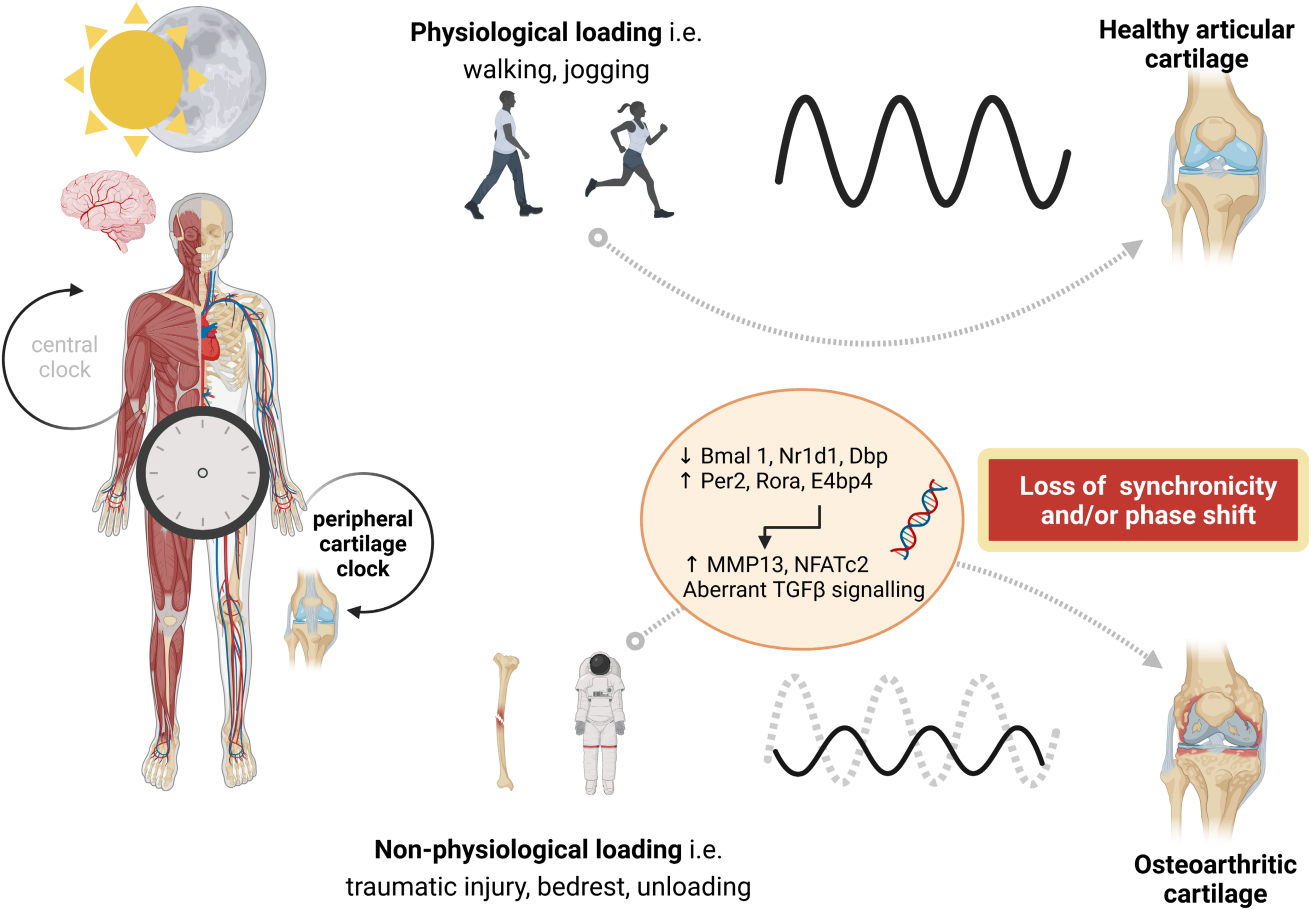
Schematic overview depicting the influence of physiological loading on the chondrocyte clock in articular cartilage. Application of non‐physiological loads for example traumatic injury, non‐weight‐bearing interferes with the cartilage clock, impacting its rhythmicity (synchronization and/or phase alignment); sustained dysregulation of the cartilage clock can induce expression of matrix degrading enzymes, proinflammatory cytokines, and inflammatory mediators leading to development of osteoarthritis (created in Biorender).

Mechanical loading can regulate peripheral clocks to promote homeostatic tissue turnover. Although cartilage clock disruption contributes to OA pathology and a pivotal risk factor for OA is abnormal loading, there is still a knowledge gap in the putative interplay or involvement of mechanical forces and cartilage clock rhythmicity. Specifically, it is still largely unknown how mechanical load influences the cartilage clock, leading to several questions: the upstream mediators which transduce the biomechanical stimulus to prime chondrocyte clocks, how physiological or non‐physiological loads influence it—is there a threshold above or below which circadian rhythmicity is altered or disrupted, at what point does mechanically‐induced disruption in circadian rhythmicity lead to an irreversible homeostatic shift promoting a catabolic phenotype. As demonstrated by the studies reviewed here, exercise has been shown to synchronize and/or entrain peripheral clocks, suggesting the capability of exercise in resetting circadian rhythms in these tissues. Future research needs to investigate whether application of physiological loads to injured or OA cartilage resets the chondrocyte clock to prevent an irreversible catabolic shift; this would have significant implication for strategies involved in cartilage tissue engineering and regenerative medicine. Emerging knowledge that mechanical stimulation can synchronize stem cell clocks (Rogers et al., 2017; Vago et al., 2022) could also be beneficial to prime cells for the intended tissue repair response; this is particularly relevant for mechanically synchronizing chondroprogenitor cells to induce chondrogenesis for cartilage regenerative approaches. Other therapeutic strategies which could benefit from improved knowledge of cartilage clock mechano‐regulation include consideration of the most appropriate time to exercise or physiotherapy for OA patients to induce optimal responses. This under‐explored area of *“chronobiology meets mechanobiology”* in articular cartilage presents an exciting research avenue where future endeavors should focus on understanding the influence of physiological and non‐physiological loading on chondrocyte circadian rhythmicity and how that impacts cartilage homeostasis (Figure 2). Collectively, both existing data (as reviewed here) and future research targeting some of the currently unanswered questions identified above will undoubtedly assist in devising mechanisms to harness the circadian nature of cartilage chondrocytes and its mechano‐regulatory potential to provide new and effective regenerative medicine opportunities.

## AUTHOR CONTRIBUTIONS

LD—manuscript drafting, final approval; CEH—conception of study, manuscript revision, final approval; EJB–conception of study, manuscript drafting and revision, final approval.

## CONFLICT OF INTEREST STATEMENT

The authors declare that there are no conflicts of interest.
